# Therapeutic T cells induce tumor-directed chemotaxis of innate immune cells through tumor-specific secretion of chemokines and stimulation of B16BL6 melanoma to secrete chemokines

**DOI:** 10.1186/1479-5876-5-56

**Published:** 2007-11-14

**Authors:** Hauke Winter, Natasja K van den Engel, Dominik Rüttinger, Jürgen Schmidt, Matthias Schiller, Christian H Poehlein, Florian Löhe, Bernard A Fox, Karl-Walter Jauch, Rudolf A Hatz, Hong-Ming Hu

**Affiliations:** 1 Department of Surgery, LMU Munich, Klinikum Grosshadern 81377 Munich, Germany; 2 Laboratory of Molecular and Tumor Immunology, Robert W. Franz Cancer Research Center, Earle A. Chiles Research Institute, Providence Portland Medical Center, Portland, Oregon, USA; 3 Departments of Molecular Microbiology and Immunology; Environmental and Biomolecular Systems and OHSU Cancer Institute, Oregon Health and Science University, Portland, Oregon, USA; 4 Department of Radiation Oncology and OHSU Cancer Institute, Oregon Health and Science University, Portland, Oregon, USA; 5 Laboratory of Tumor Immunobiology, Robert W. Franz Cancer Research Center, Earle A. Chiles Research Institute, Providence Portland Medical Center, Portland, Oregon, USA

## Abstract

**Background:**

The mechanisms by which tumor-specific T cells induce regression of established metastases are not fully characterized. In using the poorly immunogenic B16BL6-D5 (D5) melanoma model we reported that T cell-mediated tumor regression can occur independently of perforin, IFN-γ or the combination of both. Characterization of regressing pulmonary metastases identified macrophages as a major component of the cells infiltrating the tumor after adoptive transfer of effector T cells. This led us to hypothesize that macrophages played a central role in tumor regression following T-cell transfer. Here, we sought to determine the factors responsible for the infiltration of macrophages at the tumor site.

**Methods:**

These studies used the poorly immunogenic D5 melanoma model. Tumor-specific effector T cells, generated from tumor vaccine-draining lymph nodes (TVDLN), were used for adoptive immunotherapy and in vitro analysis of chemokine expression. Cellular infiltrates into pulmonary metastases were determined by immunohistochemistry. Chemokine expression by the D5 melanoma following co-culture with T cells, IFN-γ or TNF-α was determined by RT-PCR and ELISA. Functional activity of chemokines was confirmed using a macrophage migration assay. T cell activation of macrophages to release nitric oxide (NO) was determined using GRIES reagent.

**Results:**

We observed that tumor-specific T cells with a type 1 cytokine profile also expressed message for and secreted RANTES, MIP-1α and MIP-1β following stimulation with specific tumor. Unexpectedly, D5 melanoma cells cultured with IFN-γ or TNF-α, two type 1 cytokines expressed by therapeutic T cells, secreted Keratinocyte Chemoattractant (KC), MCP-1, IP-10 and RANTES and expressed mRNA for MIG. The chemokines released by T cells and cytokine-stimulated tumor cells were functional and induced migration of the DJ2PM macrophage cell line. Additionally, tumor-specific stimulation of wt or perforin-deficient (PKO) effector T cells induced macrophages to secrete nitric oxide (NO), providing an additional effector mechanism for T cell-mediated tumor regression.

**Conclusion:**

These data suggest two possible sources for chemokine secretion that stimulates macrophage recruitment to the site of tumor metastases. Both appear to be initiated by T cell recognition of specific antigen, but one is dependent on the tumor cells to produce the chemokines that recruit macrophages.

## Background

The adoptive transfer of tumor-specific T cells can mediate tumor regression and long-lasting anti-tumor immunity in the B16BL6-D5 (D5) murine melanoma model [1-3]. Examination of the effector mechanisms responsible for tumor regression showed that effector T cells deficient in perforin, IFN-γ or both perforin and IFN-γ were still able to cause tumor regression [2-4]. However, if TNF was neutralized in T cells deficient in perforin and IFN-γ significant tumor regression was no longer observed [4]. These results suggest a triad of molecules that can "compensate" for one another and define a minimal functional requirement for T cells to mediate tumor regression in this model. Further, these findings led us to question how cells deficient in perforin, a molecule essential for direct cytotoxicity, induced tumor regression.

Immunohistochemical analyses of pulmonary metastases identified macrophages to be a dominant component of the cellular infiltrate following adoptive transfer of therapeutic T cells and IL-2. These same infiltrates were not seen in progressively growing tumors or animals treated with IL-2 alone [2]. Since this occurred within 24 hours of adoptive T cell transfer we considered that these infiltrating cells might play a role in tumor regression. But what triggered the influx of monocytes? Numerous reports have identified a correlation between a tumor-specific type 1 cytokine profile and therapeutic T cells [1,5-8]. Since the CC-chemokines MCP-1 (CCL2), MIP-1α, (CCL3), MIP-1β (CCL4), and RANTES (CCL5), as well as the CXC chemokine IP-10 (CXCL10), and MIG (CXCL9) were found to be associated with a type-1 T cell response [9-11], we posited that there may be a link between expression of these chemokines, macrophage infiltration of tumor metastases and therapeutic efficacy.

Chemokines are small polypeptide signaling molecules that bind to and activate a family of seven transmembrane G protein-coupled chemokine receptors. Chemokines are responsible for the selective recruitment and activation of mononuclear cells [12,13], and they induce the directed migration of leukocytes, stimulate their adhesion and trans-endothelial migration [14,15]. Thus, we considered chemokines might be responsible for the infiltration of monocytes into pulmonary metastases. Here we investigated chemokine release following the encounter of therapeutic T cells with the specific tumor cells they recognized and how these interactions might promote tumor destruction.

## Methods

### Mice

Female C57BL/6J (wt), C57BL/6 -PFP^tm1Sdz ^(PKO) mice were purchased from the Jackson Laboratory (Bar Harbor, ME) and maintained in a specific pathogen-free environment. Mice were generally 8 to 12 weeks old at the time of experimentation. Recognized principles of laboratory animal care were followed (Guide for the Care and Use of Laboratory Animals, National Research Council, 1996). All animal protocols were approved by the Earle A. Chiles Research Institute Animal Care and Use Committee.

### Cell lines

D5 is a poorly immunogenic subclone of the spontaneously arising B16BL6 melanoma. An early passage of the original tumor was subcloned by limiting dilution. D5-G6 is a stable clone originally transduced with a murine GM-CSF retroviral MFG vector [16]. D5-G6 cells secrete approximately 200 ng/ml/10^6 ^cells/24 h GM-CSF. MPR-4 is a transformed prostate tumor cell line generated from a C57BL/6 mouse [17]. MCA-310 (H-2^b^) is a methylcholanthrene-induced sarcoma and DJ2PM is a transformed macrophage cell line (generously provided by A.V. Palleroni, Hoffmann-La Roche Inc., Nutley, NJ, USA), both were generated in C57BL/6 mice [18].

### Reagents

The 145-2c11 hybridoma (anti-CD3) was a gift from J. A. Bluestone (University of Chicago, Chicago, IL). Recombinant murine IFN-γ was purchased from Pepro-Tech (London, UK) and recombinant human TNFα was provided by Cetus (Emeryville, CA). Recombinant human IL-2 was provided by Chiron Inc. (Emeryville, CA). The anti-CD4 (GK1.5, TIB-207), anti-CD8 (2.43, TIB-210), anti-NK1.1 (PK136, HB-191), anti-Mac-1 (M1/70, TIB-128), and rat anti-mouse IFN-γ (IgG1, R4-6A2) hybridomas were obtained from American Type Culture Collection (Manassas, VA). Ascites were prepared in DBA/2 mice primed with pristane and immuno-suppressed by injection with 200 mg/kg cyclophosphamide. Purified anti-granulocyte Ab, GR-1, FITC- and PE-labeled isotype control rat IgG, hamster IgG, and mAb against CD3, CD4, and CD8 were purchased from PharMingen (San Diego, CA).

### Culture conditions

Lymphocytes and tumor cells were cultured in complete medium (CM), which consisted of RPMI 1640 containing 0.1 mM non-essential amino acids, 1 mM sodium pyruvate, 2 mM L-glutamine, and 50 mg/ml of gentamicin sulfate (Bio Whittaker, Walkersville, MD.). This was further supplemented with 50 mM 2-mercaptoethanol (Aldrich, Milwaukee, WI), and 10% fetal bovine serum (GIBCO BRL, Grand Island, NY). Tumor cells were harvested 2-to 3 times per week by brief trypsinization (Trypsin, Bio Whittaker, Walkersville, MD) and maintained in T-75 or T-150 culture flasks.

### Generation of effector T cells from tumor vaccine-draining lymph nodes (TVDLN)

D5-G6 tumor cells were harvested by trypsinization, washed twice with HBSS and resuspended at 2 × 10^7 ^cells per ml. One million D5-G6 tumor cells were injected s.c. into both hind and fore flanks of wt and PKO mice. Eight days following vaccination the draining superficial inguinal and axillary lymph nodes were harvested and TVDLN isolated. Effector T cells were generated by culturing TVDLN at 2 × 10^6 ^cells per ml in CM with 50 ul of a 1:40 dilution of 2C11 ascites (anti-CD3) as described previously [1]. After 2 days of activation, T cells were harvested and expanded in CM containing 60 IU rhIL-2/ml for 3 additional days. Effector T cells were then harvested, washed twice in HBSS, counted and used for adoptive transfer and in vitro assays.

### Adoptive Immunotherapy and Immunohistochemical analysis of tumor-bearing lungs

Experimental pulmonary metastases were established by i.v. inoculation of 2 × 10^5 ^D5 tumor cells. Mice with 3-day established pulmonary metastases received 90,000 IU IL-2 alone or together with the adoptive transfer of wt or PKO effector T cells (35 × 10^6^). Twenty-four hours later mice were killed, and frozen sections of the lungs were prepared. Tissue sections were blocked with avidin and biotin and then stained with a control rat IgG, anti-CD4, anti-CD8, anti-NK1.1, anti-Mac-1, or anti-Gr-1 Abs (specified above). Sections were washed and incubated with biotin-labeled goat anti-rat IgG, washed, and incubated with the Vectastain ABC reagent (Vector Laboratories, Burlingame, CA). Slides were developed using diaminobenzidine solution (Vector) and counterstained with hematoxylin. Images were acquired using a micropublisher CCD camera and Q capture 2.18.0 software (Q imaging, Burnaby, BC, Canada).

### RT-PCR

D5 cells were cultured in CM and different concentrations of rhTNF-α (3.5, 35, 350 U/ml) or rmIFN-γ (2, 10, 20 ng/ml) for 2, 4, or 12 hours. To characterize tumor-specific expression of chemokine, effector T cells (4 × 10^6^) were cultured alone, with immobilized anti-CD3, with a syngeneic, but unrelated sarcoma, MCA-310, or with D5 (4 × 10^5^) for 6 hours. RNA was extracted from D5 and effector T cells using the RNeasy mini kit (Qiagen, Hilden, Germany). cDNA was synthesized using 2 ug of total RNA oligo p(dT)_15 _(Roche, Mannheim, Germany) and murine MLV reverse transcriptase (Life Technologies, Karlsruhe, Germany) according to a standard protocol. Semi-quantitative PCR for different chemokines was performed as published elsewhere with slight modifications [19]. The typical amplification reaction contained 1× PCR buffer, 200 mM dNTP, 100 pg of each 3' and 5' primers, and 1 U of Taq DNA polymerase in 50 μl reaction volume. The nucleotide sequences of the primers used in the amplification reactions are depicted in Table 1. After 1 min of predenaturation at 95°C, thermocycling conditions were 30 sec denaturation at 95°C, 30 sec annealing at 60°C, and 45 sec extension at 72°C. Twenty amplification cycles were performed for HPRT, 25 cycles for the other gene amplifications.

**Table 1:** Primer sequences of chemokines and control genes

mRNA	primer sequence:*
Mip 1α	**F: **ATG AAG GTC TCC ACC ACT GCC CTT G **R: **GGC ATT CAG TCC AGG TCA GTG AT
Mip 1β	**F: **GTT CTC AGC ACC AAT GGG CTC TGA **R: **CTC TCC TGA AGT GGC TCC TCC TG
KC	**F: **CGG AAT TCG CCA CCA GCC GCC TG **R: **CGT CTA GAC TTT CTC CGT TAC TTG G
IP-10	**F: **CCT ATC CTG CCC ACG TGT TG **R: **CGC ACC TCC ACA TAG CTT ACA
RANTES	**F: **CAT CCT CAC TGC AGC CGC CC **R: **CCA AGC TGG GTA GGA CTA GAG
MIG	**F: **ATG AAG TCC GCT GTT CTT TTC C **R: **TTA TGT AGT CTT CCT TGA ACG AC
MCP-1	**F: **CTC ACC TGC TGC TAC TCA TTC **R: **GCT TGA GGT GGT TGT GGA AAA
TNF-α	**F: **GTT CTA TGG CCC AGA CCC TCA CA **R: **TAC CAG GGT TTG AGC TCA GC
GP-100	**F: **AAA TGC CAA CCA CAG AGG TC **R: **CAA GCA TTA TGG TGT CGG TG
HPRT	**F: **GTT GGA TAC AGG CCA GAC TTT GTT G **R: **GAG GGT AGG CTG GCC TAT AGG CT

*F = forward = sense = 5'-, R = reverse = antisense = 3'-primer sequence

### Real time reverse transcription-PCR

A two-step reverse transcription-PCR (RT-PCR) protocol was used for the detection of CCR1, CCR5 and 18S rRNA. Total cellular RNA was isolated from 1 × 10^6 ^DJ2PM cells according to the manufacturer's instructions (TriReagent, SIGMA-Aldrich). Genomic DNA contamination was removed by treatment with RNase-free DNase (Invitrogen, Karlsruhe, Germany) for 15 minutes at 37°C. An aliquot of 1 μg RNA was reverse transcribed with an oligo (dT) 15 primer using the avian myeloblastosis virus reverse transcriptase (AMV, first-strand synthesis kit for RT-PCR, Roche Diagnostics). Complete absence of genomic DNA was confirmed by control reactions without AMV. Real-time PCR was performed by the LightCycler technology (Roche Diagnostics, Mannheim, Germany) with SYBR Green fluorescence. PCR amplification was performed with the LightCycler Fast Start Reaction Mix SYBR Green I, including a three-segment amplification protocol: initial denaturation at 95°C for 10 minutes followed by 30 cycles of amplification of 10 seconds at 95°C, 4 seconds at 55°C for CCR1 and CCR5 or 62°C for 18S rRNA, and 5 seconds at 72°C. Primer sequences are listed in Table 2.

**Table 2:** Primer sequences of chemokine receptors and control genes

mRNA	primer sequence:*
CCR1	**F: **CCA CTC CAT GCC AAA AGA CT **R: **ACT AGG ACA TTG CCC ACC AC
CCR5	**F: **CGA AAA CAC ATG GTC AAA CG **R: **GTT CTC CTG TGG ATC GGG TA
18SrRNA	**F: **CGG CTA CCA CAT CCA AGG AA **R: **GCT GGA ATT ACC GCG GCT

*F = forward = sense = 5'-, R = reverse = antisense = 3'-primer sequence

All primers were purchased from Metabion (Martinsried, Germany). Amplified products were separated on a 2% TAE agarose gel.

### Measurement of cytokines

After activation and expansion effector T cells were washed, resuspended in CM + IL-2 (60 IU/ml) and seeded at 4 × 10^6^/well in a 24 well plate (Corning Costar, Cambridge MA.). The cells were either cultured without further stimulation or stimulated with either 2 × 10^5 ^D5, or MCA 310 tumor cells, or immobilized anti-CD3. Supernatants were harvested after 24 hours and tested for chemokines by ELISA. Similarly, D5 tumor cells were cultured alone or with rmIFN-γ or rhTNF-α at the concentrations specified and supernatants harvested after 24 hours. Release of MIP-1α, MIP-1β, KC, MCP-1 and RANTES was determined in duplicate by ELISA using commercially available reagents (B&D Biosciences, PharMingen, San-Diego, CA). The concentration of chemokines in the supernatant was determined by regression analysis.

### Detection of CCR expression by flow cytometry

DJ2PM cells (10^6^) were incubated for 24 h in CM media alone or in CM media containing 20 ng recombinant murine IFN-γ or 20 ng recombinant murine TNF-α (both from B&D Biosciences, PharMingen, San-Diego, CA), a combination of both or supernatants of 24 h anti-CD3 stimulated wt effector T cells. DJ2PM cells were harvested, washed and stained either with the anti-CCR5-Biotin mAb or the corresponding IgG2c-Biotin control (B&D Biosciences, PharMingen) for 20 min at 4°C. Cells were then washed and stained with streptavidin-PE-Texas-Red (PE-TR; Caltag, now Invitrogen, CA, USA) for 20 min at 4°C. DJ2PM cells were washed and CCR5 expression was analyzed using the Dako CyAN flow cytometer (Dako Colorado Inc., CO, USA).

### Chemotaxis assay

Either 10^5 ^D5 melanoma cells, 10^6 ^effector T cells or both D5 and effector T cells were plated in CM into the bottom chamber of a 24- transwell plate (Corning Costar, Cambridge MA.). Control wells contained medium, medium with 1 ng/ml each of recombinant MIP-1α and MIP-1β (R&D) or effector T cells cultured in wells precoated with anti-CD3 (10 μg/ml). DJ2PM cells were incubated with Carboxyfluorescein Diacetate, Succinimidyl Ester (CFDA-SE, 0.5 μM in PBS) according to the supplier's instructions (Invitrogen). In blocking experiments, DJ2PM were incubated at 37°C for 90 min with pertussis toxin (0.05 μg/ml, 0.5 μg/ml, or 5 μg/ml, Sigma) at 2.5 × 10^6 ^cells/ml. DJ2PM (3.5 × 10^5 ^per well) were washed in CM and resuspended in 250 ml prewarmed and pH-adjusted CM into the top chamber of the transwell plates. After 20 hours of incubation at 37°C, 5% CO_2_, all cells that had migrated through the filter to the lower chamber were collected by a short trypsinization, washed 2 times in FACS buffer (PBS, 0.5% BSA, 0.02% Na-Azid), blocked with Fc-γ III/II Receptor (2.4G2, BD Biosciences, San-Diego, CA) and stained with anti-CD11b antibody (BD Biosciences). The number of macrophages (i.e., the number of CD11b/CFDA-SE double-positive stained cells) that migrated into a well was determined by flow cytometry. Measurement was performed in the presence of propidium iodide to exclude dead cells.

### Nitric oxide detection

Elicited peritoneal macrophages were generated in C57BL/6 mice by injecting 5 ml thyoglycolate ip for 3 consecutive days. Seven days after the first injection the animals were killed by CO_2_, and macrophages were harvested from the peritoneal cavity by washing with HBSS, resuspended in CM and allowed to adhere to petri dishes for 1 hour. Non-adherent cells were removed and adherent cells were harvested by scraping. The cells were washed with HBSS and 1 × 10^5 ^macrophages plated with 4 × 10^6 ^effector T cells. The cells were either cultured without further stimulation or stimulated by either 2 × 10^5 ^D5, MPR-4 tumor cells, or anti-CD3. Supernatants were harvested after 4 hours and the release of NO was determined in duplicate using GRIES reagent (G4410, Sigma, St. Louis, MO). The concentration of NO in the supernatant was determined by regression analysis.

### Statistical analysis

The significance of differences in the number of migrating macrophages and cytokine secretion was determined using a Student unpaired *t*-test. Two-sided p values of < 0.05 were considered to be significant.

## Results

### Adoptive immunotherapy with wt or PKO effector T cells promotes rapid infiltration of Mac-1^+^ and GR-1^+^ cells

**Figure 1:**
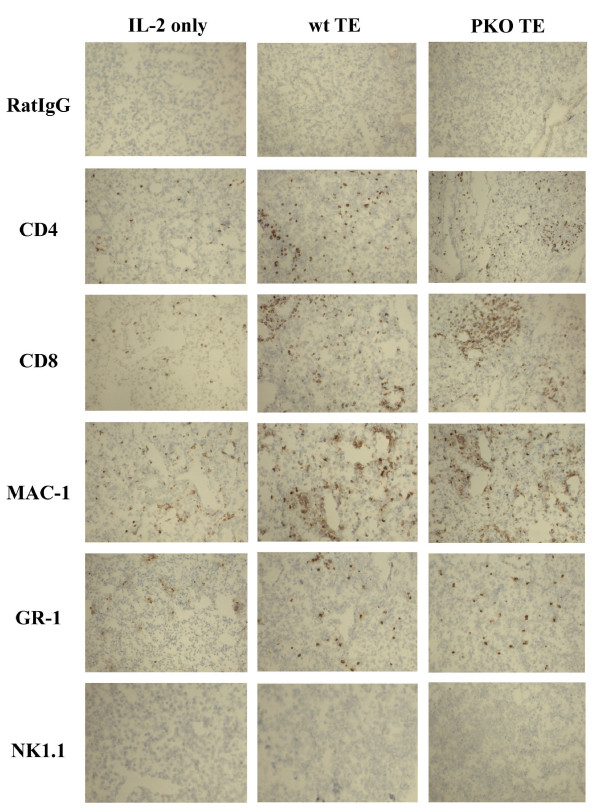
Immunohistochemistry of tumor-bearing lungs of mice 24 hours after immunotherapy with either IL-2 alone (left panel) or IL-2 and effector T cells from wt mice (middle panel) or PKO mice (right panel). Lungs were frozen and sections were cut and stained with control IgG, anti-CD4, anti-CD8, anti-NK1.1, anti-MAC-1, or anti-GR-1 Abs.

Lungs from mice bearing 4-day established pulmonary metastases were obtained 24 hours after adoptive transfer of tumor-specific T cells and frozen sections were stained with mAbs against CD4, CD8, NK1.1, Mac-1, and GR-1. Control mice did not receive T cells, but all animals received treatment with IL-2. Lungs from tumor-bearing control animals treated with IL-2 alone contained relatively few infiltrating cells (Figure 1, left panel). As reported by us previously, there were substantial increases in the number of CD4^+^, CD8^+^, Mac-1^+^, and GR-1^+^ cells in the tumor-bearing lungs of mice that received wt (middle panel) effector T cells (Figure 1) [3]. However, comparing these results to those obtained after adoptive transfer of PKO (right panel) effector T cells there were no apparent differences in the level of infiltrating CD4^+^, CD8^+^, Mac-1^+^, and GR-1^+^ cells (Figure 1). NK1-1^+ ^cells were not found in any lung section, although the Ab did stain a positive control tissue on the same slide (data not shown).

These results document, that following the adoptive transfer of tumor-specific T cells and IL-2, cells of the innate immune system, particularly macrophages and granulocytes, infiltrate pulmonary metastases. While it is generally considered that macrophages serve a "mopping-up" role following immune-mediated cell death, we wondered how PKO effector T cells which lack the capacity for rapid killing (4 hr cytotoxicity) and require longer periods of cell-cell contact to induce cell death were apparently inducing the same rapid influx of Mac-1^+^ and GR-1^+^ cells into the lung [2].

### Chemokine expression by effector T cells

**Figure 2:**
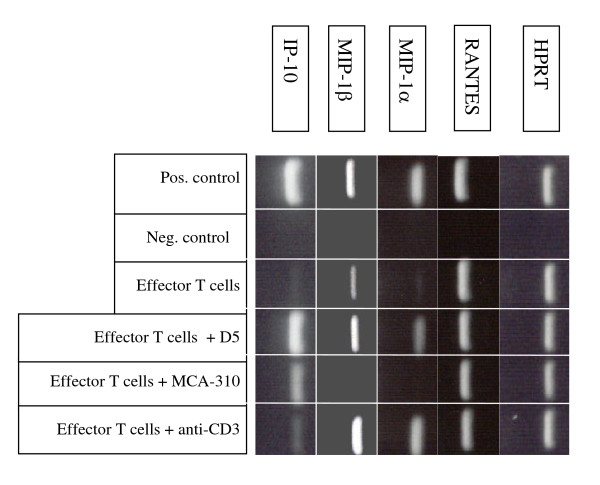
MIP-1α MIP-1β RANTES and IP10 mRNA-expression by effector T cells. Effector T cells were cultured alone (unstimulated control), with D5 (specific tumor stimulation), with a syngeneic but unrelated tumor MCA-310 (specificity control), or with anti-CD3. T cells were harvested 6 h later to determine MIP-1α MIP-β, RANTES and IP10 mRNA expression. RNA isolated from the macrophage cell line J774 was stimulated with IFNγ (20 ng/ml) and LPS (10 μg/ml) for 4 h and used as a positive control for chemokine message. Negative controls included water instead of cDNA. Hypoxanthine phosphoribosyltransferase 1 (HPRT) is a commonly used reference gene that was used as a control.

The local infiltration of cells from the innate immune system following adoptive transfer of wt or PKO effector T cells suggested the local production of chemokines to attract cells to the tumor microenvironment. Thus, we investigated whether the adoptively transfered tumor-specific T cells might be the source of chemokines responsible for the infiltration of innate immune cells. Tumor-specific effector T cells from TVDLN, activated with anti-CD-3 and expanded in IL-2, were examined for the expression of mRNA for various chemokines. Chemokine expression in T cells stimulated with specific tumor (D5) or syngeneic, but unrelated tumor (MCA-310) was compared to expression in unstimulated T cells or T cells stimulated with anti-CD3. Anti-CD3 induced high levels of message for MIP-1α and MIP-1β. RANTES expression was high in unstimulated effector T cells and did not change following stimulation with anti-CD3 or specific tumor cells. In contrast, IP-10, MIP-1α and MIP-1β message, which were weakly expressed in unstimulated effector T cells, showed increased expression following stimulation with specific tumor cells or anti-CD3 (Figure 2).

### Tumor-specific secretion of chemokines

**Figure 3:**
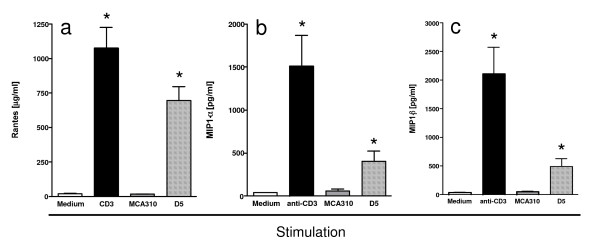
Secretion of RANTES (a), MIP-1α (b) and MIP-1β (c) by effector T cells. Effector T cells generated from D5-G6 TVDLN wt mice were assessed for tumor-specific chemokine release. T cells (4 × 10^6^/ml) were cultured alone, with anti-CD3, MCA-310 (2 × 10 ^5^/ml), or with D5 (2 × 10^5^/ml). Supernatants were harvested 18–24 h later for quantification of RANTES, Mip-1α and Mip-1β. Cytokine release into the supernatant was determined by ELISA using a standard kit. Data are presented as the mean ± SE of three independent experiments. The limit of detection for RANTES, MIP-1α and MIP-1β was 10 pg/ml. * p < 0.05

To confirm that cytokines were actually secreted, supernatants from effector T cells stimulated with D5, the syngeneic, but unrelated sarcoma (MCA-310) or anti-CD3 were assayed for RANTES, MIP-1α and MIP-1β. T cells cultured alone (unstimulated), or stimulated with MCA-310, did not secrete significant levels of these chemokines (Figure 3). However, T cells stimulated with their "specific" tumor (D5), secreted significant (p < 0.05) amounts of all three chemokines. Since effector T cells generated in response to vaccination with D5-G6 exhibit a highly polarized type 1 cytokine profile. These results are consistent with reports correlating RANTES, MIP-1α and MIP-1β expression with type 1 T cells [20,21]. KC and MCP-1, which are not secreted by T cells, could not be detected in the supernatants of unstimulated or anti-CD3 stimulated effector T cells. However, low levels of both cytokines were detected in the supernatants of effector T cells stimulated with D5 tumor cells (data not shown), suggesting that these chemokines were being secreted by the tumor cells following interaction with tumor-specific type 1 effector T cells.

### Chemokine expression and secretion by D5 tumor cells

**Figure 4:**
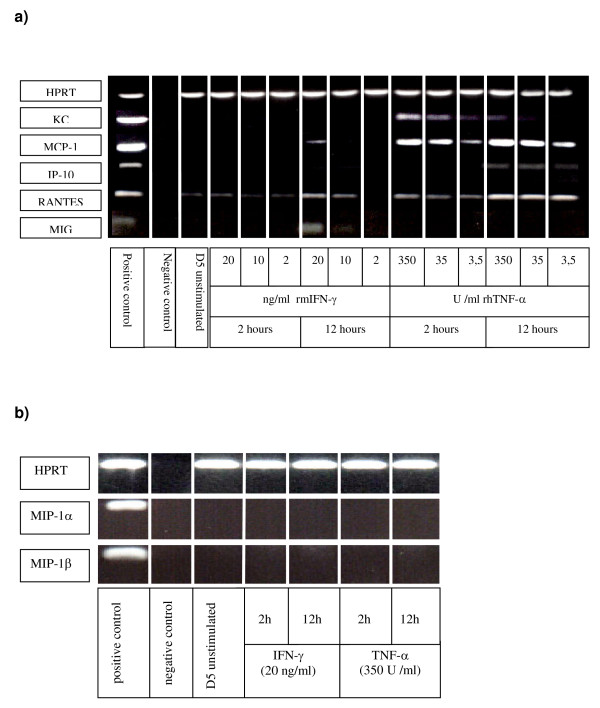
**a) **mRNA-expression of KC, MCP-1, IP-10, RANTES and MIG in unstimulated and stimulated D5 tumor cells. D5 tumor cells were cultured alone or stimulated with IFN-γ (2, 10, 20 ng/ml) or TNF-α (3.5, 35, 350 U/ml) for 2 and 12 hours. Tumor cells were harvested and mRNA expression determined by RT-PCR. **b) **Chemokine mRNA-expression of MIP-1α and MIP-1β in unstimulated and stimulated D5 tumor cells. D5 tumor cells were cultured alone or stimulated with IFN-γ (20 ng/ml) and TNF-α (350 U/ml) for 2 and 12 hours. D5 tumor cells were harvested and mRNA expression determined by RT-PCR. RNA isolated from the macrophage cell line J774 was stimulated with IFNγ (20 ng/ml) and LPS (10 μg/ml) for 4 h and used as a positive control. Negative controls included water instead of cDNA.

**Figure 5:**
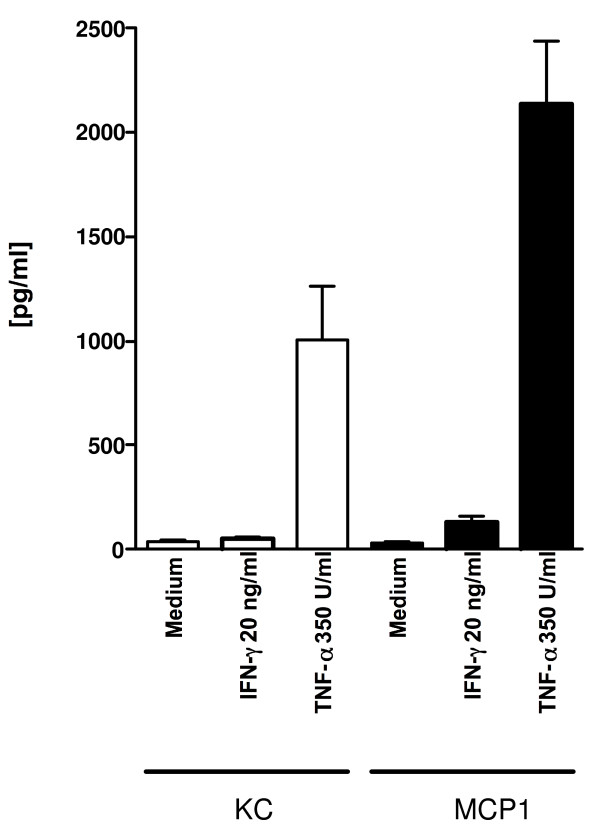
D5 tumor cells were cultured with TNF-α (350 U/ml) or IFN-γ (20 ng/ml) for 24 hours and culture supernatants tested for secretion of KC and MCP-1 by ELISA (mean of 2 experiments).

Although there is evidence that tumor cells constitutively express and secrete chemokines that can attract macrophages [22-24], we did not observe macrophages around the D5 pulmonary metastases in untreated animals with established pulmonary metastases or in animals treated with IL-2. After the chemokines expressed by effector T cells were identified, we investigated whether chemokines were also expressed by D5. Interestingly, message for RANTES was expressed constitutively at low levels whereas KC, MCP-1, IP-10, MIG, MIP-1α and MIP-1β were not expressed (Figures 4a, b). However, while message for RANTES was detected, D5 cultured in CM did not secrete substantial amounts of RANTES (data not shown). Since therapeutic tumor-specific effector T cells secrete the type-1 cytokines IFN-γ and TNF-α [2,4], we determined whether chemokine expression by D5 was induced following stimulation with IFN-γ and TNF-α. Twelve hours of incubation with rmIFN-γ (20 ng/ml) induced mRNA expression for MCP-1 and MIG and upregulated mRNA expression for RANTES. Incubation of D5 with rhTNF-α (3.5 U/ml) induced mRNA expression of KC and MCP-1 after only 2 hours. After 12 hours of incubation with rhTNF-α the expression of MCP-1 and RANTES increased further and mRNA for IP-10 was detected. While D5 tumor cells cultured without cytokine did not secrete RANTES, KC or MCP-1, ELISA confirmed that D5 tumor cells cultured with TNF-α (350 U/ml) secreted all 3 chemokines (Figure 5 and data not shown).

### Co-culture of T cells and tumor cells stimulate macrophage chemotaxis

**Figure 6:**
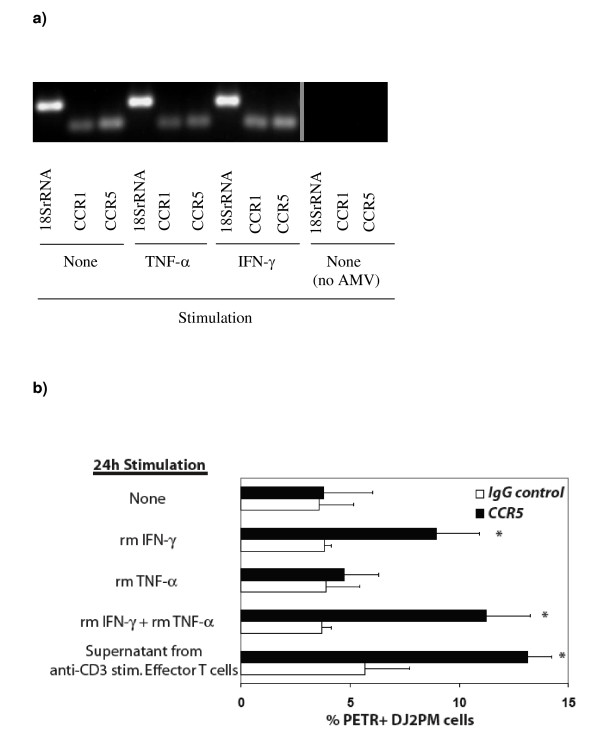
**a) **CCR1 and CCR5 mRNA-expression in unstimulated and stimulated DJ2PM macrophages. DJ2PM macrophages were cultured alone or stimulated with IFN-γ (20 ng/ml) or TNF-α (35 U/ml) and mRNA expression determined by RT-PCR. **b) **Mean percentage of DJ2PM cells expressing CCR5 receptor as determined by flow cytometric analyses. Cells were stained with specific mAb or isotype control. Graph shows the percentage of PE-TR positive DJ2PM cells (mean ± SEM). Histograms with * exhibit significantly (*p = 0.01) greater expression of CCR5 compared to isotype controls. Data are a summary of 3 consecutive experiments.

**Figure 7:**
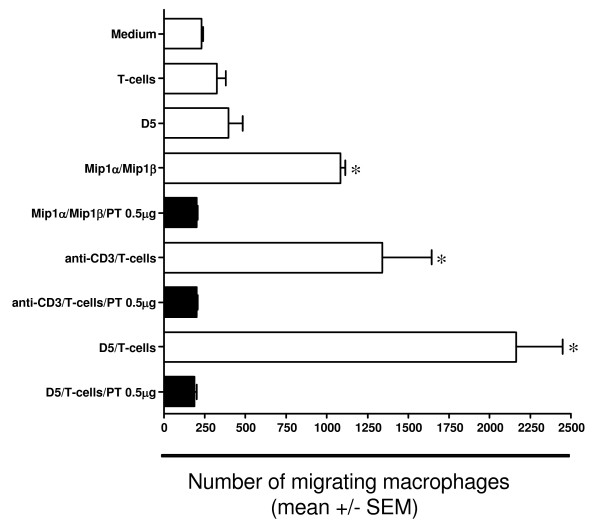
Chemotaxis-assay of DJ2PM macrophages either pretreated with (black columns) or without (white bars) PTx (0.5 g/ml). Migration towards serum control, MIP-1α/MIP-1β, effectotr T cells (T_E_), D5 or D5 co-incubated with T_E _was determined after 4 hours. MIP-1α/MIP-1β, T_E_, D5 and T_E _co-cultured with D5 were plated into the bottom chamber of a 24 well transwell plate for 12 hours. Subsequently 3.5 × 10^5 ^peritoneal macrophages (DJ2PM) were added to the top chamber for 4 hours. The number of macrophages that migrated into the lower chamber was determined by flow cytometry as the percentage of CD11b+/CDFA-SE+ cells. Data are presented as the mean ± SE of three independent experiments (* p < 0.05).

To confirm the interaction of T cells and tumor cells released substances that were biologically active and capable of recruiting monocytes/macrophages we developed a chemotaxis assay. We assessed the DJ2PM macrophage cell line for mRNA expression of chemokine receptors for MIP-1α and MIP-1β, CCR1 and CCR5. The DJ2PM macrophage cell line expresses message for both CCR1 and CCR5 (Figure 6a). We next examined expression of the CCR1 and CCR5 by flow cytometry. While the rabbit polyclonal anti-human CCR1 is known to cross-react with mouse CCR1, background staining using two different sources of rabbit anti-CCR1 was high and experiments were not informative (data not shown). However, staining with the anti-CCR5 mAb detected a significant percentage of DJ2PM cells expressing CCR5 following culture with IFN-γ, IFN-γ + TNF-α, or anti-CD3-stimulated T cell supernatants (Figure 6b). To show that chemokines secreted by effector T cells were chemotactic for this macrophage cell line, we performed a chemotaxis assay with DJ2PM macrophages in the upper well of a transwell chamber and with D5, effector T cells, or a combination of both, plated into the lower transwell chamber. The capacity of the DJ2PM macrophage cell line to migrate in response to chemokines was confirmed by migration of macrophage into a lower chamber containing MIP-1α and MIP-1β (positive control, Figure 7). Unstimulated effector T cells plated into the lower chamber did not induce the migration of macrophages into the lower transwell chamber. While D5 tumor cells induced some migration of macrophages into the lower chamber, this increase did not reach statistical significance. However, a significant increase in macrophage migration into lower wells was observed when effector T cells and D5 tumor cells were cocultured in the lower chamber (p < 0.05). A significant increase in DJ2PM cell migration into the lower chamber was also noticed when effector T cells placed in the lower chamber were stimulated with anti-CD3 (p < 0.05) (Figure 7). An increased migration of macrophages was also observed after stimulation of D5 tumor cells with IFN-γ (10 ng/ml) or TNF-α (35 U/ml) (data not shown).

For further investigation of the nature of the chemotactic activity produced following anti-CD3 stimulation of tumor-specific T cells or coculture of D5 with D5-specific effector T cells, we treated DJ2PM macrophages with PTx (0.5 mg/ml). Chemokines bind to seven-transmembrane-domain receptors that are coupled to heterotrimeric G_i _proteins. It is well established that chemokine-induced migration is completely inhibited by treatment of the cells with PTx, which inhibits Ga_i _and Ga_0 _proteins [25]. In these studies, incubation of DJ2PM with either 0.05 μg/ml, 0.5 μg/ml or 5 μg/ml of PTx completely inhibited the migration towards anti-CD3 stimulated T cells and T cells stimulated with D5, suggesting that heterotrimeric G-proteins are involved in the signal transduction pathway of the T cell-induced DJ2PM migration (Figure 7 and data not shown). Together, these data document that D5-specific effector T cells with therapeutic function in adoptive transfer studies are capable of inducing chemotaxis of a macrophage cell line if stimulated with specific tumor or anti-CD3. They also demonstrate that D5 melanoma cells exposed to TNF-α or IFN-γ can lead to the release of chemokines that are biologically active and capable of inducing chemotaxis of a macrophage cell line.

### Activation of macrophages by effector T cells

**Figure 8:**
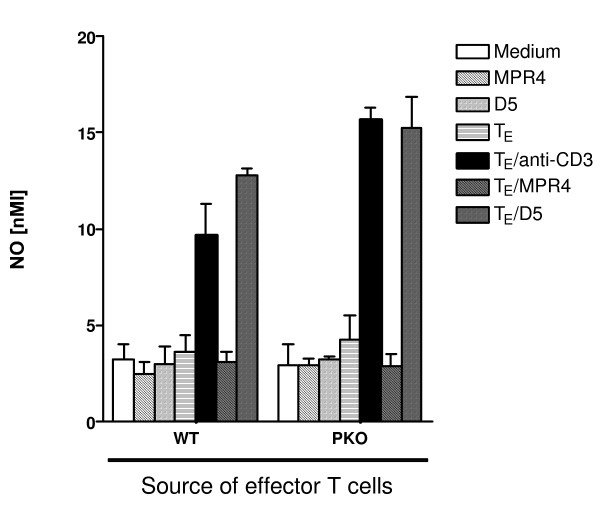
NO release of thioglycolate-elicited macrophages. Thioglycolate-elicited peritoneal macrophages and effector T cells (T_E_) were plated into a 24-well plate and co-cultured without or with D5, MPR-4 (a syngeneic but unrelated tumor), or anti-CD3. Macrophages and tumor cell co-cultures without the addition of effector T cells was an additional control. Supernatants were harvested after 4 hours and the release of NO determined in duplicate by GRIES reagent using commercially available reagents. Data are presented as the mean of three independent experiments (SEM) * p < 0.05

To investigate whether macrophages can be activated by effector T cells, thioglycolate-elicited peritoneal macrophages from wt C57BL/6 mice were cultured with effector T cells generated from wt and PKO mice. Unstimulated effector T cells from wt and PKO mice did not induce the secretion of NO from macrophages. Furthermore, secretion of NO was not observed when peritoneal macrophages were co-cultured with D5 or the syngeneic tumor MPR-4, or after stimulation of effector T cells with MPR-4. However, significant NO secretion was observed when macrophages were incubated with wt or PKO effector T cells activated with anti-CD3 or D5 (Figure 8).

## Discussion

A number of groups, including ours, have reported that T cells from mice deficient in specific effector molecules can still mediate tumor regression in adoptive transfer experiments [2-4]. Although tumor regression in these models is clearly T cell-mediated, the specific mechanism(s) by which tumors are destroyed have not been fully characterized. Further, the potential contributions of components of the innate immune system to tumor regression following adoptive T cell immunotherapy have not been studied. Usually, one thinks about the initial activation of the innate immunity, which leads to the activation of an adaptive immune response. The converse may also be true.

Hung et al. demonstrated that CD4^+ ^T cell-dependent immunity elicited by vaccination with a GM-CSF gene-modified B16 tumor was dependent on both macrophages and granulocytes [26]. Based on this report and our own demonstration of substantial infiltrates of macrophages and granulocytes in pulmonary metastases twenty-four hours following adoptive transfer of tumor-specific T cells, we initiated studies to explain these observations.

Macrophages play a role in both, innate and adaptive immune responses. They process and present antigens and act by orienting adaptive responses toward a type I or type II phenotype, as well as by expressing specialized and polarized effector functions [27-30]. Mantovani et al. showed that macrophages, like T cells, can be segregated into subsets that differ in their receptor expression pattern, cytokine and chemokine production, and effector functions [21]. Cytokines and microbial products determine the polarization of mononuclear phagocytes. IFN-γ alone or in concert with microbial products (LPS) or other cytokines (e.g. TNF-α), polarizes macrophages towards a type-1 (M1) phenotype. M1 macrophages typically have a high capacity to present antigen, secrete large amounts of interleukin-12 (IL-12) and IL-23, and produce NO and reactive oxygen intermediates [31]. M1 macrophages were shown to be tumoricidal [21,32-36]. In contrast, M2 polarized macrophages are characterized by an IL-10^high ^and IL-12_low _phenotype. They are induced by immune complexes (IC), LPS and the type-2 cytokines IL-4 and IL-13 [29,37]. Macrophages, spontaneously infiltrating tumor, were shown to be M2 polarized macrophages promoting tumor progression [28,38].

Macrophages are attracted towards sites of infection and tumor deposits by chemokines. Chemokines can also polarize macrophages and influence the tumor environment in ways that can promote or retard tumor growth [21]. Therefore, we investigated whether tumor-specific T cells with therapeutic activity could attract macrophages by the coordinated, tumor-specific release of chemokines. Unstimulated effector T cells did not secrete chemokines. In contrast, anti-CD3 and tumor-specific stimulation of effector T cells resulted in secretion of MIP-1α, MIP-1β and RANTES which have been positively correlated with a type-1 immune response [9-11]. RANTES, MIP-1α, and MIP-1β are strongly chemotactic for mononuclear cells and DC [39]. Further, we note that message for these chemokines was detectable in T cells as early as 6 hours following stimulation with anti-CD3, suggesting that monocyte infiltration to tumor sites could be triggered rapidly following adoptive T cell transfer. We confirmed that some component of the secreted molecules was biologically active as tumor-specific stimulation of effector T cells or D5 exposed to IFN-γ or TNF-α induced the chemotaxis of the DJ2PM macrophage cell line in vitro. Further, preincubation of DJ2PM with PTx completely inhibited macrophage migration towards effector T cells, suggesting that heterotrimeric G-proteins are involved in the signal transduction pathway responsible for the chemokine-induced macrophage migration [25].

Furthermore it was shown earlier, that MIP-1α, MIP-1β and RANTES influence the polarization of T cells towards a type- phenotype [11]. In the D5 model we appreciate that the endogenous immune response that occurs following adoptive transfer is critical for the cure and development of long-lived immunity in treated animals [40]. The influx of macrophages with an M1 profile may facilitate long-lived immunity by promoting the priming and expansion of endogenous tumor-specific type1 T cells. Besides the chemotactic effect on macrophages and its potential to influence the polarization of T cell responses there is evidence that MIP-1α can directly affect the metastatic potential of melanoma cells. Van Deventer et al. showed that MIP-1α-transfected B16 F10 melanoma cells formed significantly fewer pulmonary metastases as compared to tumor cells that did not express MIP-1α [41]. Whether RANTES, MIP-1α and MIP-1β are direct growth inhibitors of D5 tumors remains to be determined.

In contrast to type-1 cytokines secreted by effector T cells, which have been positively correlated with tumor rejection, the role of chemokines constitutively secreted by tumors is somewhat controversial [20,42-44]. D5 expressed low levels of RANTES and KC constitutively, however, very few macrophages were identified in untreated D5 pulmonary metastases. In contrast to our observation, Nesbit et al. and Haghnegahdar et al. showed that melanoma cells secrete low levels of MCP-1 inducing the accumulation of tumor-associated macrophages and promoting tumor progression [23,45]. Furthermore melanoma cells were shown to express IL-8, Gro-α, Gro-β and MCP-1, all of which are implicated in tumor growth and progression [45].

Our own data shows that chemokine secretion by D5 was markedly upregulated after stimulation with the type-1 cytokines, IFN-γ and TNF-α, expressed by therapeutic tumor-specific effector T cells. This observation is consistent with findings by Sonouchi et al. who found a significant increase of KC, MCP-1 and IP-10 mRNA expression in RENCA kidney cancer cells after stimulation with IFN-γ, IFN-α and IL-2 [46]. Together, these findings support a model where tumor-specific T cells recognize tumor cells in situ and provide an initial trigger, delivering chemokines and cytokines at the tumor site. In addition to chemokines directly recruiting granulocytes and monocytes, the type 1 cytokines also induce tumor metastases to secrete additional chemokines, amplifying the recruitment of innate effector cells. In this model it is the combination of events that lead to the infiltration of monocytes and granulocytes resulting in T cell-mediated tumor destruction. In other models the same cytokine cascade may lead to the production of immune suppressing factors (IL-10, TGF-β, and VEGF), resulting in immune suppression and tumor progression. This explanation may shed light on the apparent controversy regarding the role of inflammation in tumor progression/regression.

Monocyte chemoattractant protein-1 (MCP-1, CCL2) is expressed in inflammatory conditions, is a chemoattractant for monocytes and T lymphocytes, and plays a pivotal role in autoimmune diseases [47]. We were able to show that MCP-1 expression by D5 is strongly induced following culture with effector T cells or the inflammatory cytokines IFN-γ and TNF-α. Therefore, one explanation for the influx of macrophages is that effector T cells, following recognition of specific tumor, induce the expression and secretion of high levels of MCP-1 by D5 tumor cells which in turn results in the influx of macrophages, eventually aiding and/or leading to tumor destruction. In contrast to this explanation, Peng et al. showed that neutralizing MCP-1, secreted by the MCA-205 sarcoma, improved the therapeutic efficacy of tumor-specific effector T cells in mediating regression of established pulmonary metastases [48]. Future studies will be required to delineate the role of MCP-1 in the D5 melanoma model.

Besides MCP-1, IP-10, and MIG expression by D5 were also upregulated following exposure to TNF-α and IFN-γ, respectively. Arenberg et al. were able to show that intra-tumoral injection of IP-10, or MIG led to reduced tumor growth in a SCID mouse model of NSCLC and postulated an anti-angiogenic effect of these 6C-kines [44]. This was confirmed by Palmer et al. who demonstrated that M1 polarized macrophages secrete the CXC chemokines IP-10 and MIG which were shown to have anti-angiogenic properties and to inhibit tumor progression [49]. Interestingly no effect of 6C-kines on the growth of a human lung cancer cell line (A549) or on the infiltration of leukocytes into established tumors of SCID-mice was observed [44]. Tannenbaum et al. showed that antibodies against IP-10 and MIG blocked IL-12 inducible tumor regression and decreased the amount of tumor-infiltrating T cells in the murine kidney cancer tumor model RENCA [50]. Thus it can be speculated that tumor regression induced by IP-10 and MIG is mediated by the release of IL-12 that in turn promotes the induction of tumor-specific T cells with a type 1 cytokine profile. This positive feedback loop may help to explain why the Renca tumor cell line is so strongly immunogenic. It has to be evaluated further whether this is also true in our melanoma tumor model.

Future studies will need to provide a more quantitative and qualitative analysis of inflammatory cell infiltrates. Current efforts are directed at using multiparameter flow-based methods to characterize monocytes and macrophages. Such characterization of the innate immune cells infiltrating the tumor site may provide additional insights into the effector mechanisms that are operational during T cell-mediated tumor destruction.

## Conclusion

We show that tumor-specific T cells with therapeutic efficacy secrete chemokines in response to tumor-specific stimulation. While this seems obvious, studies dissecting the mechanism of T cell-mediated tumor regression have focused on perforin, IFN-γ, TNF and Fas-L. We are unaware of a report that considers the influx of innate cells as a possible effector mechanism in T cell-mediated regression of pulmonary metastases following adoptive immunotherapy. Instead, this influx of macrophages is commonly considered a response to "mop-up" dead cells. Here we show that the chemokines secreted by T cell – tumor co-cultures are chemotactic for macrophages in vitro and may explain the rapid accumulation of macrophages at the site of pulmonary metastases following adoptive immunotherapy with therapeutic T cells. Furthermore, tumor-specific effector T cells were able to change the chemokine expression pattern of D5 tumor cells such that they secreted chemokines that were chemotactic for macrophages in vitro. This provides an "amplification" pathway to augment the chemokines secreted by T cells. Finally, we show that co-cultures of effector T cells and tumors can promote activation of macrophages that secrete NO. The tumoricidal activity of NO secreted by macrophages may account for some of the therapeutic efficacy following adoptive T cell immunotherapy. These observation provide additional insights into the multitude of mechanisms potentially involved in T cell-mediated tumor regression as well as a possible explanation for the heterogeneity in clinical responses observed among patients on clinical adoptive immunotherapy trials. Future studies will need to characterize whether failure to express chemokines following exposure to IFN-γ and/or TNF-α represents a new class of resistance mechanisms employed by tumors to escape immune destruction. Finally, these observations may also prove useful in explaining why inflammation can be associated with tumor progression in some models and tumor regression in others.

## Abbreviations

Complete medium (CM), macrophage inflammatory protein-1 alpha (MIP 1α/CCL3), macrophage inflammatory protein-1 beta (MIP 1β/CCL4), monocyte chemoattractant protein (MCP-1/CCL2), regulated on activation, normal T cell expressed and secreted (RANTES/CCL5), interferon gamma inducible protein 10 (IP-10/CXCL10), monokine induced by interferon-gamma (MIG/CXCL9), keratinocyte-derived chemokine (KC), perforin knock-out mouse (PKO), pertussis toxin (PTx)

## Competing interests

The author(s) declare that they have no competing interests.

## Authors' contributions

HW performed the adoptive immunotherapy studies and initial ELISA studies for T cell secretion of chemokines and activation of macrophages to secrete NO. HW also drafted the manuscript. NE performed the ELISA assays for chemokine expression by tumor cells, established the chemotaxis assay, discussed the data and reviewed the manuscript. MS performed the chemotaxis assays.

DR and CP performed PKO adoptive transfer studies and participated in the design of the studies. JS performed molecular studies of chemokine expression by tumor cells. CP correlated the immunohistochemistry data, performed CCR5 flow studies and participated in the design of the studies. FL reviewed the manuscript and discussed the data.

The initial studies were performed in the lab of BF. BF, together with HW and HH conceived and initiated the studies to determine whether T cells were secreting molecules that might be responsible for migration and activation of macrophages. BF helped edit the manuscript. KJ provided support and participated in the design of the study. RH directed the Laboratory where the molecular studies were performed, participated in study design and obtained support for the studies. HH conceived the studies to evaluate whether type 1 cytokines induced expression of chemokines by D5 tumor cells and participated in the design of all studies. HH helped draft the manuscript. All authors read and approved the final manuscript.
